# Domain Adaptation with Contrastive Simultaneous Multi-Loss Training for Hand Gesture Recognition

**DOI:** 10.3390/s23063332

**Published:** 2023-03-22

**Authors:** Joel Baptista, Vítor Santos, Filipe Silva, Diogo Pinho

**Affiliations:** 1 Department of Mechanical Engineering (DEM), Institute of Electronics and Informatics Engineering of Aveiro (IEETA), University of Aveiro, 3810-193 Aveiro, Portugal; vitor@ua.pt; 2 Department of Electronics, Telecommunications and Informatics (DETI), Institute of Electronics and Informatics Engineering of Aveiro (IEETA), University of Aveiro, 3810-193 Aveiro, Portugal; fmsilva@ua.pt; 3 Bosch Termotecnologia, S.A., EN 16-km 3.7-Cacia, 3800-533 Aveiro, Portugal; diogo.pinho@pt.bosch.com

**Keywords:** human–robot interaction, hand gesture recognition, distribution shift, transfer learning, contrastive learning, multi-loss simultaneous training

## Abstract

Hand gesture recognition from images is a critical task with various real-world applications, particularly in the field of human–robot interaction. Industrial environments, where non-verbal communication is preferred, are significant areas of application for gesture recognition. However, these environments are often unstructured and noisy, with complex and dynamic backgrounds, making accurate hand segmentation a challenging task. Currently, most solutions employ heavy preprocessing to segment the hand, followed by the application of deep learning models to classify the gestures. To address this challenge and develop a more robust and generalizable classification model, we propose a new form of domain adaptation using multi-loss training and contrastive learning. Our approach is particularly relevant in industrial collaborative scenarios, where hand segmentation is difficult and context-dependent. In this paper, we present an innovative solution that further challenges the existing approach by testing the model on an entirely unrelated dataset with different users. We use a dataset for training and validation and demonstrate that contrastive learning techniques in simultaneous multi-loss functions provide superior performance in hand gesture recognition compared to conventional approaches in similar conditions.

## 1. Introduction

Hand gestures are an important aspect of human communication serving several purposes, such as enhancing spoken messages, signaling intentions, or expressing emotions. Driven by technological advances, the process of classifying meaningful hand gestures, known as hand gesture recognition (HGR), has received increasing attention in recent years [1,2]. The major application areas of gesture recognition include sign language translation, human–machine interaction, medical rehabilitation, and virtual reality. HGR systems also target robotic applications using a variety of input devices, among which stands out color cameras, depth sensors, or gloves with embedded sensors. In this context, the ability of robots to recognize hand gestures seems to be very promising for progress in human–robot collaboration (HRC), since they are simple and intuitive to produce by a human partner [1,3]. HRC aims to explore new technologies and methods allowing humans and robots to coexist and cooperate in the same environment in order to improve the overall efficiency of the task, distribute the workload, and/or reduce the risk of injury [4,5,6,7].

In HRC, it is imperative to develop solutions that are trustworthy, safe, and efficient [8], which means establishing a robust communication channel capable of real-time practical utility. The use of voice communication in noisy industrial environments can lead to errors, miscommunication, and safety risks. In these circumstances, it may be preferable to explore non-verbal communication, such as static hand gestures [9,10]. However, HGR is an inherently challenging task due to the complex non-rigid properties of the hand, such as its shape, color, and orientation. Even more importantly, vision-based HGR systems must be robust to variations in lighting conditions, cluttered environments, complex backgrounds, and occlusions [11,12,13], which can be even more accentuated in industrial environments. In collaborative scenarios, we must add the challenges that arise in terms of real-time processing and safety requirements (e.g., the recognition system must be designed to only respond to the pre-determined intentional gestures).

As a result, researchers are constantly developing new techniques and algorithms to improve the accuracy and robustness of HGR systems. In line with the trend of learning systems, convolutional neural networks (CNNs) have been successfully applied in image recognition tasks, while recurrent neural networks (RNNs) are a natural choice in recognizing gestures from videos [14,15]. One solution for HGR is to include a hand segmentation module as the first stage of the pipeline [16,17]. The process of separating the hand from the background allows the recognition model to focus on the relevant information of the input image while reducing the impact of variations in the background or lighting conditions. However, in industrial collaborative scenarios, hand segmentation is difficult (or even not feasible), requiring sophisticated segmentation algorithms and high-resolution images. More recently, methods based on deep learning have demonstrated robust results by training the gesture recognition model in the context of the entire image [18,19].

A significant number of studies devoted to static gestures use transfer learning as a solution to the data challenge and training constraints. They use as a baseline the knowledge from a deep model previously trained on a large labeled dataset (e.g., ImageNet [20]) and re-purpose it for another task. However, the assumption that the training and test datasets are drawn from the same distribution rarely holds in a practical situation due to a domain shift. For example, hand gestures can vary significantly across users and illumination conditions, leading to differences in the statistical properties of the two datasets.

A visual recognition system with its model trained on a dataset obtained from a couple of users under specific illumination conditions, and tested on a different dataset based on different users and illumination conditions, will most likely be affected by a domain shift. This may lead to poor generalization performance and model failure in real-world applications. In order to address this problem, domain adaptation techniques can be used to improve the generalization performance of the visual recognition model on the target dataset [21,22,23,24]. These techniques aim to align the statistical properties of the source and target domains by reducing the distribution shift between them. Adversarial training and fine-tuning are two common approaches to minimize the difference between the feature distributions of the source and target domains while maximizing the prediction accuracy [25,26].

This paper proposes a domain adaptation technique for hand gesture recognition in human–robot collaboration scenarios. The proposed approach is based on a two-headed deep architecture that simultaneously adopts cross-entropy and a contrastive loss from different network branches to classify hand gestures. The main goal is to shed light on the impact of supervised contrastive learning (SCL) on the generalization ability of the trained deep model when faced with a distribution shift. For this purpose, the study contributes with a new RGB annotated dataset of hand gestures extracted in a cluttered environment under changing lighting conditions. The training data were obtained from a single subject (the source domain), while the test data involved three new users (the target domain).

In order to investigate the effectiveness of the proposed approach, we compare our results against two baselines. The first baseline corresponds to a conventional transfer learning approach in which a pre-trained model is fine-tuned on the source domain, aiming to demonstrate a significant drop in performance when applied to a target domain. The second baseline uses the traditional supervised contrastive learning in which the classification problem is separated into two phases. First, a pre-trained model is used as an encoder to learn good representations of the input data using a contrastive loss. Then, a classifier is trained on top of the learned representations (frozen network) using a standard cross-entropy loss. In order to evaluate the generalization performance of the HGR model on different datasets, a cross-dataset evaluation is conducted using evaluation metrics such as accuracy, precision, recall, and F1-score. To the best of our knowledge, this is the first attempt to apply contrastive learning in static hand gesture recognition in order to align the statistical properties of two datasets.

The remainder of the paper is organized as follows: Section 2 presents related work on vision-based hand gesture recognition. Section 3 describes the experimental setup underlying the study, including the new dataset acquisition pipeline. Section 4 details the transfer learning methods and the deep architectures used. Section 5 focuses on the experiments conducted and on the comparative performance evaluation carried out. Finally, the conclusions and future work are provided in Section 6.

## 2. Related Work

HGR is a well-studied problem in computer vision and has been applied to a variety of applications, including HRI. In recent years, deep learning (DL) techniques have shown significant improvements in the accuracy and robustness of HGR systems. The problem of HGR can be divided into five main steps: image acquisition, hand detection, feature extraction, classification, and output gesture [27]. Recently, DL has shown great performance in solving these steps, mainly by using convolutional neural network (CNN)-based methods.

In general, the majority of hand gesture recognition studies focus on dynamic gestures for sign language recognition (SLR). In [28], the authors propose a method based on spatiotemporal features, using CNN and LSTM configurations to extract features from the hand, face, and lips in order to classify the dynamic gesture. This approach was tested in the AUTSL [29] dataset with good performance. Another work tested in this dataset was the approach proposed in [30], which explored a multi-modal solution with RGB and depth images. This approach extracted spatiotemporal features using 3DCNNs. The authors also used a pre-trained whole-body pose estimator to obtain body landmarks used to further improve the model’s performance. Although this solution has great results in AUTSL, the accuracy decreases when tested in WLASL-2000. Although these studies with dynamic gestures, which represent the state-of-the-art in SLR, have good performance in constrained conditions, they still lack robustness and generalization capabilities in order to be used in HRI scenarios. For this reason, the state-of-the-art of HGR for HRI focuses on improving static gesture recognition in unforeseen conditions, in order to establish a communication channel with minimal message errors.

In [18], the authors use a faster R-CNN model for HGR to pass commands to a robot. This model could perform hand detection and classification simultaneously on four different gestures. The detected coordinates of the hands were used to define a decision threshold, improving the classifier’s accuracy. The authors in [19] also propose a DL approach for simultaneous hand detection and classification based on YOLOv3. The model is trained to find the bounding box of every hand in an image and provide the most probable classification.

In another approach [31], the authors propose an HRI communication pipeline using the Machine Learning (ML) Framework MediaPipe [32] to extract relevant landmarks from the hands. These landmarks were used to produce features that are introduced in a Multilayer Perceptron (MLP) that classifies five different hand gestures. Another work [33] improves an HGR model by fusing features extracted from the image and features extracted from the hand landmarks.

In another work [34], the authors propose an approach that tackles the complexity of vision-based HGR by implementing a DL model that learned to classify gestures in segmented (black and white) and colored images. The implementation trains the model based on convolution layers and attention blocks from scratch, in an attempt of creating a robust solution specific to HGR.

In [16], the authors tackle the problem of the background by applying skin segmentation techniques before performing gesture classification. This is achieved by modeling the pixel distributions with Gaussian mixture models (GMMs). In [17], the authors also propose background removal based on skin and motion segmentation to facilitate the classification model. In [35], the segmentation was performed using the depth channel of a Kinect RGB-D camera. The hand was segmented by applying a threshold to the distance between the hand and the camera. The authors in [36] also used the depth channel of a Kinect camera for gesture segmentation; however, the classification is performed using only the depth channel. The authors implemented TL techniques in two different CNNs, AlexNet and VGG16, which classified the hand gesture in parallel. After that, the system performed a score fusion based on the output of the two neural networks. The authors in [37] also use TL by using Inception-v3 and MobileNet architectures to propose a robust system that classifies ten different gestures. A different approach to hand segmentation was used in [38], which implemented an MLP module and morphological operations to obtain the mask of the hand.

From the literature review, it is possible to conclude that vision-based DL methods are reliable approaches for HGR. However, to tackle the high level of background complexity, most solutions focus on heavy image processing prior to gesture classification. Additionally, these processing techniques tend to be based on traditional computer vision techniques, which are very context-specific and lack flexibility and generalization capacity [31]. For this reason, we explore novel DL methodologies to train deep neural network architectures to improve robustness and generalization capabilities in complex industrial environments. To achieve this, we propose to use TL techniques to reduce the resources necessary and take advantage of the knowledge acquired in similar tasks. In addition, we explore the Supervised Contrastive Learning developed in [39].

## 3. Dataset Acquisition

This section describes the dataset for this study, including the pipeline developed for online hand gesture detection and classification. This framework was also used to acquire the datasets to train and test the machine learning model.

Before acquiring a dataset to train a classification model, we searched for existing large-scale hand gesture datasets. These datasets are useful for the scientific community as they improve the efficiency and effectiveness of the ML models, and provide comparative benchmarks for new methods. We found extensive datasets with multiple classes and users, such as TheRuSlan [40], AUTSL, WLASL, LSA64 [41], and MS-ASL [42]. Although these corpora are extremely useful in general cases, they fail to provide samples that can be used to solve our particular problem formulation. This happens because, in general, the state-of-the-art HGR is focused on dynamic gestures, while the studies of HGR for HRI are conducted for static HGR. For this reason, the major contributions of datasets, including the ones mentioned before, use dynamic gestures with video samples. These datasets were also acquired on backgrounds with a lack of complex features, which could limit the generalization capabilities of the trained model.

We also found a complete dataset in Kaggle, named ASL Alphabet (ASL dataset on Kaggle at: https://www.kaggle.com/datasets/grassknoted/asl-alphabet, accessed on 3 March 2023), which is representative of the datasets available for public utility in static hand gestures. We utilized the dataset to train a CNN model with TL techniques in this dataset and tested with images taken in an unstructured environment. The accuracy of this test was very low, mainly due to the background of the ASL Alphabet dataset being simplistic while our background was complex. This experiment motivated us to perform the acquisition of a complete training dataset in our environment. After this acquisition, we trained the same CNN with TL techniques with our data and tested using the ASL Alphabet dataset, which had an accuracy of 95%. This validates our dataset to train models to predict images with simplistic backgrounds; however, these will not be the environments used in collaborative tasks with industrial robots. For this reason, this paper focuses on using our dataset with new training techniques to increase the generalization of the HGR model.

### 3.1. Image Acquisition Pipeline

**Figure 1:**
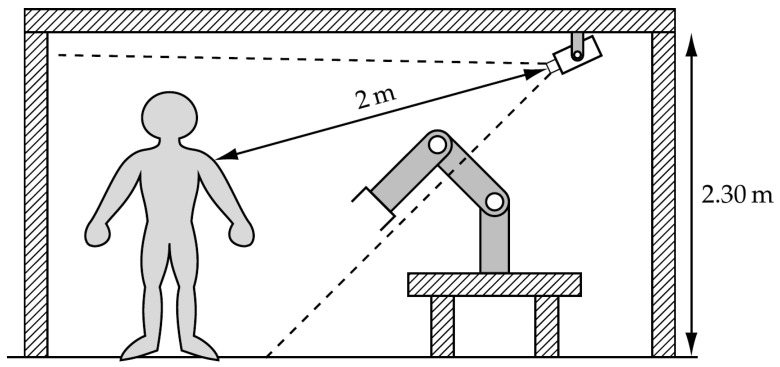
Diagram of the collaborative cell with the robotic arm and the human operator. It includes the representation of the Kinect RGB-D camera with a representation of its field of view.

The framework uses a Kinect RGB-D camera to record 480×640 RGB images. The classifier does not use the Depth channel; however, the RGB-D Kinect was chosen to allow future integration of this channel, and because of the widespread use of this device. The Kinect camera is installed in a collaborative cell with a UR10e collaborative robot [43]. The device is mounted at a height of 2.30 m, slightly tilted down to cover the working space of the operator. This area is about 2 m apart from the camera. The UR10e collaborative robot is found between the Kinect camera and the operator’s work area. This setup allows the human to be always facing the robot and the camera simultaneously. Figure 1 illustrates the physical setup described.

**Figure 2:**
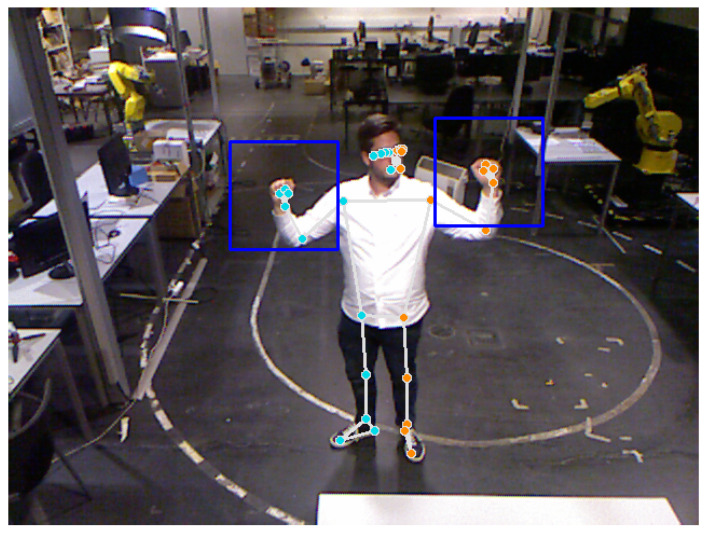
Example of user and hand gesture detection using human pose detection and tracking of the MediaPipe framework. The colored landmarks are generated by Mediapipe and the blue frames represent the region of interest for classification.

The framework was implemented in the ROS environment [44], allowing abstraction and scalability of the communication pipeline. The images are acquired using a ROS driver, which publishes the images directly to a ROS topic. After that, the hands of the operator are detected with a specific ROS node. This node uses the ML framework MediaPipe, specifically the human pose detection and tracking. The tracker predicts 33 keypoints related to the human pose, with four points for each hand. The detection node calculates the average value of the landmarks for each hand and defines a bounding box of 100×100 pixels using the average value as the bounding box center. Figure 2 shows an example of the user hand gesture detection performed by the detection node.

To reduce class variability and, henceforth, increase the classifier’s performance, the image of the right hand is flipped horizontally to appear similar to the left hand. This pipeline was used to record the training and test dataset, but it can also be used for real-time communication with the collaborative robot. The data acquisition is performed at 11 FPS.

### 3.2. Dataset Description

The main focus of this framework is to implement a reliable and robust communication pipeline for HRI in an industrial environment. For this reason, two datasets were recorded with uncontrolled background and luminosity. This implementation uses four hand gesture classes inspired by American Sign Language; the symbols chosen are the “A”, “F”, “L” and “Y” signs. These signals have the advantage of being well-known symbols with already real-world applications, implying that they are easy to use. These specific signs were chosen also because they are relatively distinct.

**Figure 3:**
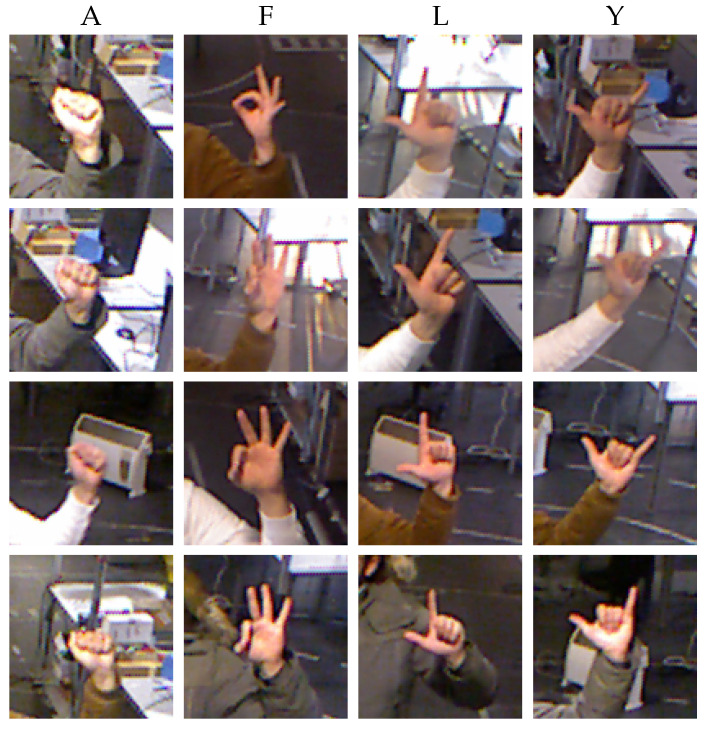
Examples of the training dataset of the four hand gesture classes in the unstructured environment and complex background. Each column is a class that is associated with an American Sign Language letter.

To test the degree of generalization of the proposed method, two datasets were recorded, which are available online (the authors’ dataset on Kaggle at https://www.kaggle.com/datasets/joelbaptista/hand-gestures-for-human-robot-interaction, accessed on 3 March 2023). The first dataset was used to train the classification model. This dataset was recorded by one user, with different luminosity and clothing, which can be seen in Figure 3.

**Figure 4:**
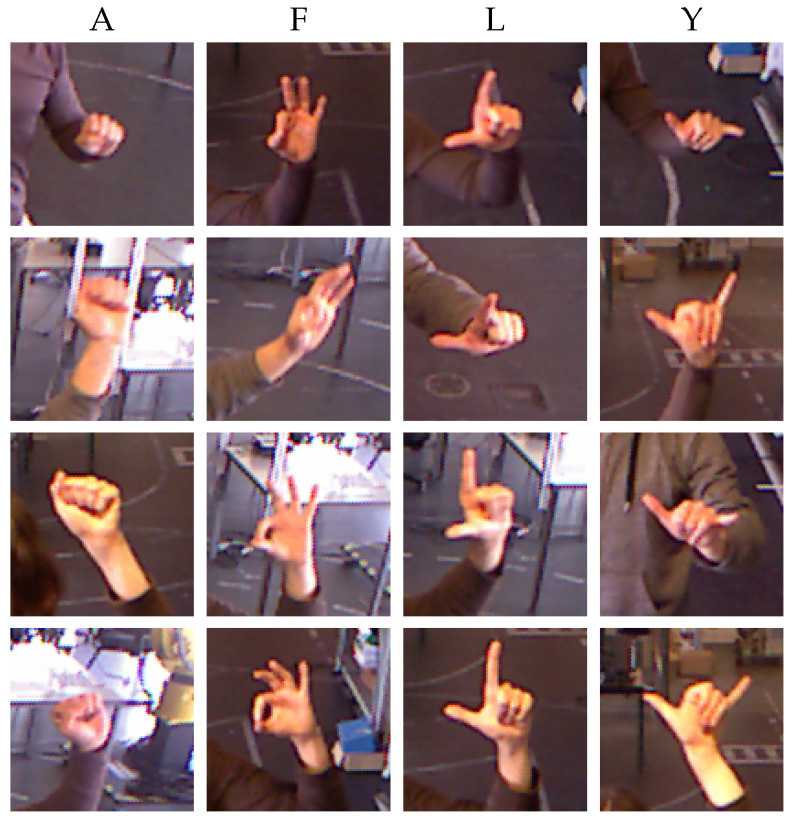
Examples of the test dataset with three different persons and acquired at different times of the day in relation to the training dataset. Each column is a class that is associated with an American Sign Language letter.

The second dataset is used to test the model. This multi-user test dataset was recorded with three persons who were not included in the training dataset. The recording was performed on a different day and at a different time of the day, resulting in variation in luminosity. Figure 4 shows some samples that constitute the multi-user test dataset.

Table 1 shows the distribution of samples among all classes. Although the dataset is small when compared to large-scale datasets, it has a distribution of samples per class similar to other static hand gesture datasets used in HGR for HRI [16,18,31]. However, its size may limit the generalization capabilities of the classifying model. This concern has led us to acquire the dataset with a high degree of variation in the background and luminosity. In addition, we apply online data augmentation in the training phase, which further helps compensate for the reduced number of samples.

### 3.3. Data Augmentation

To increase the generalization capabilities of the ML model, we use online image augmentation. The augmentation applied random transformations to the images, intending to increase the degree of variability between samples.

In this implementation, we use the RandAugment library from PyTorch [45]. The following list shows the augmentation operations utilized:

**autoContrast**: Remaps the pixel values so the lowest value pixel becomes black and the highest value pixel becomes white.
**posterize**: Reduces the number of bits used to encode a pixel value.
**contrast**: Adjusts the contrast of an image.
**equalize**: Equalizes the histograms of an image.
**saturation**: Adjusts the saturation of the image.
**brightness**: Adjusts the brightness of the image.
**translate-x**: Translates the image by the x number of pixels.
**translate-y**: Translates the image by the y number of pixels.
**shear-x**: Distorts the image along the x-axis.
**shear-y**: Distorts the image along the y-axis.
**crop and resize**: Crops the image randomly and resizes it to the original size.

It is important to mention that the first six augmentation operations change the color space of the image, providing robustness for changes in color and luminosity. The remaining operations deform the image spatially, in an attempt to generalize the classifications to other human operators. To the previously mentioned operations, we also added random crop and resizing to accommodate for the differences in the operator’s distance to the camera.

## 4. Methodologies

This section describes the proposed contrastive domain adaptation technique for hand gesture recognition, focusing on the overall design and structure of the deep architectures in comparison. Our goal is to train a model on the source domain and then use it to make predictions on the target domain that has different characteristics, namely, different users and illumination conditions.

### 4.1. Scope and Assumptions of the Study

This study performs a comparative evaluation of three deep architectures that will be trained based on the concept of transfer learning, aiming to reduce the required resources to train the ML model. The first baseline involves fine-tuning a deep model, pre-trained on the ImageNet dataset [20], using a cross-entropy loss function (single-loss training). The second architecture introduces the contrastive learning framework applied in two phases. More concretely, the learned representations from the first phase are used as input to a classifier in the second phase. This is done by optimizing a contrastive loss function in the first phase and then using a standard cross-entropy loss to train the classifier (multi-stage, multi-loss training). Finally, the proposed architecture addresses the domain adaptation problem by considering a dual-branch head approach in which two loss functions are optimized (simultaneous multi-loss training). As explained before, the models will be trained with the source dataset extracted from a single user, while they will be tested on the target dataset based on three different users.

The main assumption of this comparative study is to use Google’s Inception-v3 [46] pre-trained model for extracting features in any of the three architectures. The idea is to leverage the knowledge already learned from the large dataset before fine-tuning the model with the source dataset. This base model is used as an image feature extractor and outputs a feature vector of 2048 elements. The assumption made in this step is that a CNN trained to achieve great performance in a very diversified dataset has learned important image patterns and features for image classification. The features extracted by the Inception-v3 convolution layers are passed through a classification module, which normally consists of an MLP. It is worth mentioning that, in a preliminary phase, we tested the performance of some other pre-trained models, such as the ResNet50 [47], by applying standard TL techniques. The tests carried out revealed that Inception-v3 performed slightly better for this specific problem and dataset. However, the techniques applied in this paper are not encoder-specific, implying that this choice should not have a large impact on the investigation.

**Figure 5:**
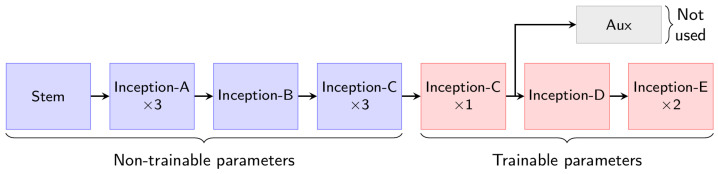
Inception-v3 architecture based on inception modules. The red modules were updated during the training phase while the blue modules were kept fixed.

The task of discriminating 1000 classes of diverse categories is certainly different from the HGR problem and, for that reason, it may be necessary to retrain some of the last convolution layers of the full base model. Figure 5 shows a simplified representation of the Inception-v3 architecture, based on the Stem and Inception modules. The lower convolutional layers are frozen as they are more likely to contain general features, while the layers closer to the output layer are fine-tuned since they are more task-specific. The objective of this work is to produce a hand gesture classification framework that can be used in unstructured industrial environments while limiting the resources and time utilized in the model’s training. For this reason, we attempted to retrain the fewest possible number of modules in the base model. We chose to retrain the last four Inception modules, as it was the smallest number of retrained modules that allowed the training to converge properly. This conclusion was empirically verified by gradually increasing the number of retrained modules.

### 4.2. Single Loss Training

**Figure 6:**
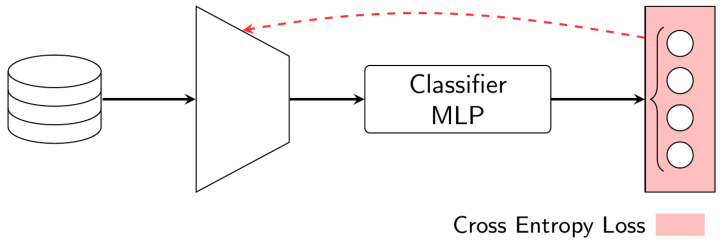
Model architecture in Single Loss Train. The implemented loss is the cross-entropy loss.

The first baseline architecture consists of the Inception-v3 pre-trained model that is re-purposed for the specific hand gesture classification task involving four classes. The single loss training (SLT) consists of the traditional approach of TL, according to the structure presented in Figure 6. This architecture uses the pre-trained model as a starting point and then trains it on the source dataset to optimize its performance. During the fine-tuning process, the weights of the last convolutional layers are updated, while the lower convolutional layers (i.e., closer to the input layer) are frozen.

This implementation replaces the last fully connected layer of Inception-v3 with an MLP. We verified experimentally that a reduction from the 2048 feature vector to 4 classes is not optimal. For this reason, the MLP is composed of four layers, where three layers reduce the feature vector from 2048 to 256, and one last layer reduces it from 2048 to 4 classes. The three first layers use the ReLu activation function, while the last layer uses the Softmax activation function. The four output classes are used to calculate the cross-entropy loss (CEL), which is used to update the trainable parameters of the base model and the classifier.

Supposedly, this process of transfer learning may not generalize well when there is a significant domain shift between the source and target datasets. Anyway, the evaluation of the model is useful as it provides a clear idea of the existing domain shift and the loss of generalization performance.

### 4.3. Multi-Stage Multi-Loss Training

**Figure 7:**
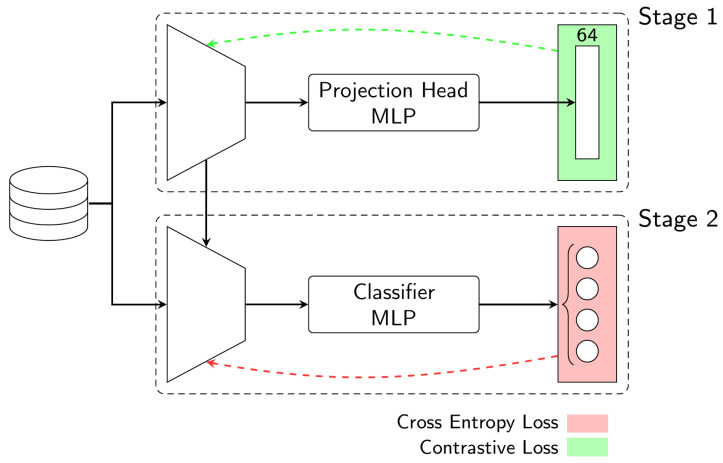
Model’s architecture in Multi-stage Multi-loss Training. In the first stage, it is used the contrastive loss and in the second stage it is used the cross-entropy loss.

Supervised contrastive learning is a technique that involves taking a pair of examples and mapping them to a common representation space. The multi-stage multi-loss training (MSMLT) consists of an adaptation of the supervised contrastive learning method in which the training process is applied separately in two stages. Figure 7 shows the architecture associated with the MSMLT method. In the first stage, the base model is trained using a contrastive loss (CL) function. This loss function encourages the base model to produce similar feature vectors for images of the same class and to move apart the feature vectors of images in different classes. The classes can be considered very similar as they are representations of the same object with different shapes. This means that the train data includes hard negative samples, which are considered as samples in different classes with similar representations or feature vectors. In these cases, supervised contrastive learning impacts the performance of the model [48]. This is performed by fine-tuning the Inception-v3 pre-trained model using the source dataset, while part of its convolutional layers are frozen.

Contrastive learning loses performance when applied to high-dimensional feature vectors. For this reason, it is usual to use a projection head that reduces the dimensionality of the feature vector, while preserving the relevant information. In this implementation, we reduce the feature vector from 2048 to 64 utilizing five linear layers with ReLu activation functions.

The output of this module is only used during the training phase, allowing the calculation of a supervised CL. This loss function compares two feature vectors, attempts to maximize the difference between them if they belong to different classes, and minimize it if they belong to the same class. With this implementation, we expect that the neural network will minimize the CL by updating its weights to produce features that are representative of hand gestures, thus ignoring the complex background of the images. The CL implementation is expressed by Equation (1),

(1) $$C_{loss}=\sum_{i\in B}\frac{-1}{p}\sum_{j\in P(i)}\log\frac{e^{(v_{i}\;\cdot \;v_{j})/\tau }}{\sum_{a\in A(i)}e^{(v_{i}\;\cdot \;v_{a})/\tau }},$$

which can be detailed as follows: while training the model, the Projection Head produces a feature vector $v_{i}$ for each image in batch *B*. Set P(i) represents the indexes of the positive samples *j* in relation to an anchor sample *i*, and it has a size of *p*. A sample is classified as positive when it belongs to the same class as the anchor. Set A(i) includes all the indexes of *B* except *i*. The exponents exhibit the dot product between two feature vectors divided by a scalar temperature parameter τ that was set to be 0.0007 during the training phase.

After that, the second stage resembles the SLT in which a classifier is trained on top of the learned representations to perform the classification of hand gestures using a standard cross-entropy loss. It is worth noting that, in this second phase, the learned representations from the first phase are frozen.

### 4.4. Simultaneous Multi-Loss Training

**Figure 8:**
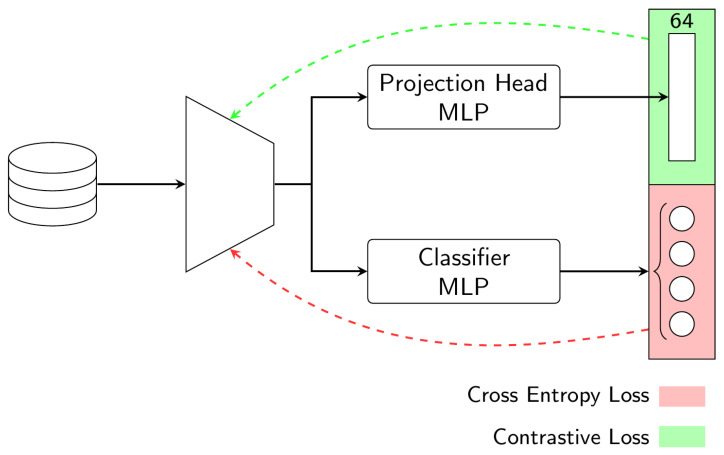
Model’s architecture in simultaneous multi-loss training. The losses used are the contrastive loss and the cross-entropy loss.

Supervised contrastive learning has been shown to be effective for domain adaptation, because it can help the model to learn features that are invariant to domain shifts [49]. Inspired by these works, we aim to show that a contrastive loss function can help improve the generalization performance by learning more robust representations that are less sensitive to distribution shifts. The idea behind the proposed contrastive domain adaptation technique is to use a network that branches twice after the encoder model (dual-branch head), allowing to train the representation model and the classification model simultaneously. Figure 8 shows the model architecture of the simultaneous multi-loss training (SMLT) approach.

On the one hand, the classifier branch uses the output of the encoder and predicts the hand gesture class label based on a softmax activation function. On the other hand, the projection head branch uses a full-connected network (MLP) to map the high-dimensional feature vector to a lower-dimensional space. The implementation of the CL is similar to the second method, where it utilizes a Projection Head MLP to reduce the feature vector from 2048 to 64 elements. This approach has been used in computer vision tasks such as image classification and object recognition [39].

As a result, two loss functions are optimized simultaneously—a cross-entropy loss for the classifier branch and a contrastive loss for the projection head branch. During training, the projection head MLP and the classifier MLP are updated by a single loss. However, the trainable parameters of the shared encoder model are updated using the two losses simultaneously. This is achieved by back-propagating the two losses sequentially in the PyTorch code, which results in adding the two gradients of the two loss functions when training the neural network model.

The goal of training with a multi-loss function is to balance the trade-off between competing objectives of accurate classification and effective feature extraction. This method aims to increase the generalization capabilities of the classification model by inducing the encoder model to produce more distinct feature vectors for each class. The difference lies in the assumption that this objective could be better achieved by optimizing these two behaviors simultaneously instead of running them separately. After training is completed, the projection head branch is removed and the model architecture will be composed of the encoder and the classifier for downstream tasks.

## 5. Results

This section describes the training and test results in the context of hand gesture classification, as presented earlier. We compare the three different approaches presented in the previous section. The training and test were performed in a computer with the processor AMD Threadripper 2850 Extreme, the graphical processing unit (GPU) NVIDIA RTX 2080TI and 128 GB of RAM.

### 5.1. Training Curves

The following graphs show the model’s accuracy and losses in each epoch for the proposed SMLT. The training curves of the other approaches were omitted because they are very similar. In our experiments, we monitored the cross-entropy loss of the validation dataset to decide when to stop the train. We stipulated that the training finished if the validation cross-entropy loss increased consecutively for five epochs. We also configured the training to save the model’s parameters that achieved the highest validation accuracy. The only exception was in the first stage of the multi-stage multi-loss train, where the validation accuracy cannot be measured. For this reason, we saved the model’s parameters that achieved the lowest validation CL.

In the different implementations, we tried to maintain the same training hyperparameters. The training was realized with a batch size of 194 images and a standard learning rate of 0.0001. The optimizer used was the ADAM [50] optimizer, which calculates adaptive learning rates for each trainable parameter. We did not implement learning rate scheduling techniques, because the adaptive learning rates produced by ADAM were sufficient to converge the model. Furthermore, the training dataset was randomly split into 60% for training, 20% for validation, and 20% for testing.

**Figure 9:**
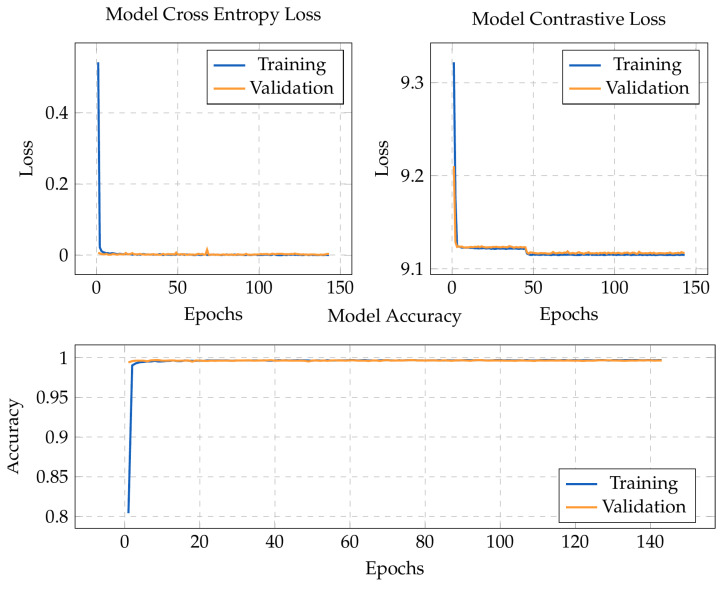
Training curves implementing the simultaneous multi-loss method. The graphs present the model’s accuracy and losses in the training and validation dataset in each epoch.

After analyzing the accuracy graph in Figure 9, we can see one of the advantages of transfer learning. The training and validation accuracy reaches 100% within a few epochs, although the batch size of 194 promotes that each epoch updates the model’s weights several times. After that, the models were tested with the test split of the training dataset, which also had results near the 100% accuracy, concluding that the models were able to learn the provided dataset with minimal overfitting. This confirms the assumption that patterns learned by the models in the ImageNet dataset were useful for hand gesture classification, given that even the retrained layers needed only a few updates for the model to fully learn to classify the training dataset. However, this is not the objective of the implementation and was rather expected by the state-of-the-art in HGR and by the fact that we are using a small dataset with four classes and only one person. The objective of this study is to increase the generalization capabilities of the model in images with complex backgrounds, and for that, it is necessary to test the proposed solution with a different dataset in different conditions.

### 5.2. Testing the Models with a New Multi-User Dataset

**Figure 10:**
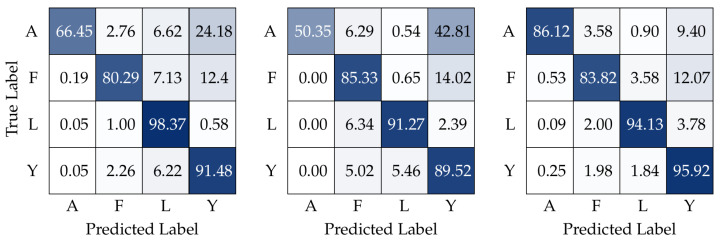
Confusion matrices of results, in percentage (%), obtained by training all three models with the single-user dataset and testing them with the multi-user dataset with different persons not present in the training dataset; the models are SLT (**left**), MSMLT **(middle**), and SMLT (**right**).

In this subsection, we present the results of testing the trained models with the multi-user dataset, in an attempt of measuring the generalization capability of the classification models. The multi-user dataset was recorded with three human operators different from the training subject, at a different time of the day. First, we present the confusion matrices (see Figure 10) obtained by testing the three models. The confusion matrices are normalized with respect to the true label.

The three confusion matrices in Figure 10 show relatively similar patterns. The first aspect to notice is that the higher values of the matrices tend to be in the matrices’ diagonals. This is the first evidence that the models acquired some degree of generalization capabilities because the diagonals represent the percentage of the True Positives. The matrices also show that the models tend to misclassify some images of the A and F classes with the Y label. This can be related to the fact that the majority of the hand in the Y class is similar to A and F, only with two fingers held up. We verified that the SMLT helped lower the wrong classification rate, by teaching the models to focus on features that more accurately separated the three classes. This confusion could also be resolved by expanding the training dataset with more examples of these classes, or adding more users; however, these approaches would not benefit our purpose, which is to study the increase of generalization capabilities with contrastive learning.

Table 2 shows the accuracy, recall, precision, and F1-score (average values) of the three approaches for the multi-user test dataset. The testing results show that simultaneous multi-loss training provides the best solution. This means that there is an advantage in using a contrastive loss in addition to the cross-entropy loss. However, it seems that for small datasets and low levels of model retraining, using the two losses separately in different training stages does not have any increases in the model’s generalization capabilities.

## 6. Conclusions

This paper proposes a domain adaptation technique for hand gesture recognition in human–robot collaboration scenarios. We defined a set of four gestures inspired by the ASL dataset, which can be used to trigger the programmed routines of the robot, allowing the human to communicate with the machine. This study was motivated by the complexity of the environment and background associated with industrial setups. In order to emulate these conditions, we created a dataset of hand gesture images in a collaborative cell with varying backgrounds and luminosity conditions to retrain the pre-trained Inception-v3 model. This source dataset was acquired by a single person. After that, we recorded a second dataset (target dataset) with three different persons to test the generalization capabilities of the deep models. This study performed a comparative evaluation of three deep architectures trained based on the concept of transfer learning.

Using knowledge acquired from the ImageNet dataset allowed the training to converge rapidly and to classify the training dataset in a few epochs. However, the actual focus of this study was the generalization capacity of the model, which was tested using the multi-user test dataset. In this testing phase, the results demonstrate that joining CEL and CL in a multi-loss training approach helps the model reach higher accuracy. In fact, this approach performed an increase of 6% in the accuracy of the model, compared to the traditional TL method of training the model only with the CEL. This shows that contrastive learning is focused on learning task-specific features, being effective to deal with the domain shift problem. However, it is important to note that applying CL separately from CEL in different training stages may not be sufficient (the results were even worse). The trade-off between accurate classification and effective feature extraction was achieved by optimizing these two behaviors simultaneously instead of running them separately.

For future work, we propose testing these different training approaches with new state-of-the-art DL models and comparing them to the results of Inception-v3. We should also increase the training and test datasets, in gestures, number of users, and size, to verify if the methodologies uphold. Lastly, we should experiment with hand gestures with industrial gloves to further simulate the industrial scenario.

## Figures and Tables

**Table 1:** Number of images, per class, of the training dataset, and the multi-user test dataset. The training dataset includes one user and the test dataset includes three users.

Dataset	A	F	L	Y	Total
Training dataset	6430	6148	5989	6044	24,611
Multi-user test dataset	4183	4277	4276	4316	17,051

**Table 2:** Evaluation metrics for testing the three different approaches in the multi-user test dataset. These values are the average of the metrics calculated for each class.

Model	Accuracy	Recall	Precision	F1
Cross-entropy loss training	84.23	84.15	86.82	84.07
Multi-stage multi-loss training	79.24	79.12	84.17	78.89
Simultaneous multi-loss training	**90.03**	**89.97**	**90.96**	**90.12**

## Data Availability

Publicly available datasets were analyzed in this study. This data can be found here: https://www.kaggle.com/datasets/joelbaptista/hand-gestures-for-human-robot-interaction, accessed on 3 March 2023.
